# Analysis of the Regional Efficiency of European Funds in Spain from the Perspective of Renewable Energy Production: The Regional Dimension

**DOI:** 10.3390/ijerph18094553

**Published:** 2021-04-25

**Authors:** Paul Mugambi, Miguel Blanco, Daniel Ogachi, Marcos Ferasso, Lydia Bares

**Affiliations:** 1 Doctoral School of International Relations and Political Science, Corvinus University of Budapest, 1093 Budapest, Hungary; paulmakunyi@yahoo.com; 2 Department of General Economics, University of Cadiz, 11002 Cádiz, Spain; miguel.blanco@uca.es; 3 Doctoral School of Economics and Regional Sciences, Hungarian University of Agriculture and Life Science, 2100 Gödöllő, Hungary; ogachdniel@gmail.com; 4 Institute of Scientific Research and Graduate School, Universidad de Lima, Lima 15023, Peru; admmarcosferasso@gmail.com

**Keywords:** energy efficiency, environment, data envelope analysis, renewable energy

## Abstract

During the 2010–2020 period, the European Union (EU) launched a growth strategy based on three fundamental pillars: smart growth, sustainable growth, and inclusive growth. Aiming to finance the projects related to these growth pillars, the EU used mainly the Rural Development Funds, the Structural Funds, those derived from the R&D Framework Program, the Trans-European Networks, and the European Investment Bank. This research aimed to determine whether the Spanish regions maintain homogeneous efficiency levels by using these resources to improve the levels of environmental quality related to renewable energies. A methodology that is frequently used by researchers in efficiency analyses was chosen, the Data Envelopment Analysis (DEA). The main findings revealed that the efficiency in the use of renewable energies is very uneven among the Spanish regions and these differences are maintained throughout the period analyzed. These results highlighted the need of changes regarding the proposed criteria for allocating European resources to finance the projects presented by each Spanish region.

## 1. Introduction

During the years 2010–2020, the European Union (EU) launched a growth strategy based on three fundamental pillars: smart growth, sustainable growth, and inclusive growth. As defined by the European Commission (EC), sustainable growth implies a new economic growth approach based on the efficient use of natural resources. It is supported both in the development of new processes and technologies and the development of smart grids. In both cases, it must be allowed the creation of a shared space of low carbon emissions, with little degradation of the environment, and avoiding the loss of biodiversity in European territory. This conceptual change in the growth model should decrease oil and gas importations by EUR 60,000 million until 2020. According to the EC’s calculations, if generating 20% of energy through renewable sources, more than 600,000 jobs could be created in the EU.

In quantitative terms, the implementation of this growth strategy should make it possible to achieve, at the end of the period and regarding environmental matters, a reduction between 20% and 30% of greenhouse gas emissions, an increase in the use of renewable energies by 20%, and an increase in energy efficiency by 20%. These goals also help to prevent climate change and improve energy sustainability [1].

The financing of these changes in this growth model has been carried out mainly through a series of EU financial instruments, such as Rural Development Funds, Structural Funds, those derived from the R&D Framework Program, Trans-European Networks, and the Bank European Investment. These resources have been distributed among the EU countries, basically attending to criteria of relative wealth. The distribution aims to reduce regional differences in Europe [2]. In the Spanish case, from an administrative sphere, a high degree of decentralization is noticed. Specifically, the Spanish State model defines 17 regions with specific competencies regarding environmental matters and according to the implementation of European Funds, i.e., their management, application, and justification of utilization.

Specific studies on territorial development in Spain showed essential differences in their growth models at the regional level [3,4,5]. For this reason, it is necessary to carry out analyses that relate, at the territorial level, the environmental changes incorporated in the growth models by using the received resources, especially those targeting renewable energy. In this way, it is possible to identify which regions are more efficient in using renewable energies thanks to the European Funds. Then, since these territorial differences have been detected, the investigation could be expanded to determine which projects have been launched in the regions and obtained the best results from this environmental aspect. Thus, the change from the economic growth model based on fossil fuels to those based on renewable energies could be expedited.

The results of this research can also be used by public administrations in order to develop regulations within their circumscriptions. These administrative bodies can incorporate the degree of environmental efficiency achieved as one additional weighting parameter in the approval of projects financed by European Funds. Equally, the projects proponents could access a national and territorial database created to share the peculiarities of the projects with the best qualification, and the region in which it has been implemented.

This type of analysis requires to define a methodology usually used by researchers in inefficiency analyses. In this manuscript, a non-parametric methodology called ‘Data Envelopment Analysis’ (DEA) and proposed by [6] was used.

In Spain, a set of measures was approved to meet the Community framework’s goals due to the new European conception of sustainable growth. Among them, the so-called Renewable Energy Plan (PER) 2011–2020 is highlighted. This plan was approved by the Agreement of the Council of Ministers of 11 November 2011, in which a series of objectives were established under Parliament’s Directive 2009/28/EC Council, of 23 April 2009. An essential financing source for the change in the growth model comes from the European Funds, as already presented. In the particular case of Spain, there is a distribution of these resources among the different regions. Thus, the Funds’ distribution is considered an instrument that enables the EU’s change towards a sustainable growth model in which renewable energies play a more significant role. Thus, the distribution of public resources among regions should consider their specificities since the undesirable situation of compliance with the objectives proposed by the PER 2011–2020 at the national level may arise. However, one limitation is that they are not achieved at the national level. Therefore, this would imply that the fulfillment of some of them would only fulfill the objectives of production of renewable energies at the national level. This process could present a feedback on how a region increasingly presents high economic and environmental growth rates to others’ detriment.

Thus, the main goal of this research is to determine if Spanish regions maintain homogeneous levels of efficiency regarding the use of European Funds to improve renewable energies as the engine of change in the growth model. For this, the following structure has been followed in this research. First, a review of the existing bibliography is made. Subsequently, an efficiency analysis model is proposed, and the variables to be used are defined. The model is then applied to the variables, which allows for a series of results to be extracted. Finally, the limitations of this study are specified, and a series of conclusions is provided.

## 2. Theoretical Background

### 2.1. Analysis of Environmental Efficiency

In recent decades, researchers have frequently addressed the environmental efficiency analysis. We highlight the investigations of [6,7,8,9,10,11,12,13,14,15,16,17,18,19,20,21,22]. In these researches, efficiency models are applied to analyze specific industries and territories. Yue et al. [6] examined the impact of multidimensional urbanization on energy–environmental efficiency using a super slack-based measure model with a stochastic impact by regression on population, affluence, and technology model. Koçak et al. [7] used a data envelopment analysis (DEA) and bootstrap DEA to study the environmental efficiency of R&D expenditures for energy efficiency, renewable energy, hydro and fuel cells, fossil energy, nuclear energy, and other power and storage technologies in OECD countries. Li et al. [8] investigated the environmental efficiency and environmental governance efficiency of China’s industrial sector from 2010 to 2017, with the methodology meta Dynamic Directional Distance Functions (DDF). Ouyang and Yang [9] analyzed regional energy and environmental efficiency with networked data envelopment analysis (DEA) and multiplicative function for 27 OECD countries. Iram et al. [10] examined the efficiency of energy for some OECD economies for the period 2013–2017 using DEA methodology. Banaeian, Omid and Ahmadi [11] examined the effective energy utilization on strawberry production in Tehran with DEA analysis. Ebrahimi and Salehi [12] used DEA constant returns to scale and variable returns to scale model to study the pattern of energy use and CO2 emission from mushroom production in Iran. Khoshnevisan et al. [13] explored energy use efficiency in greenhouse cucumber production using DEA model. [14].

Mousavi-Avval et al. [14] used DEA to determine energy use pattern for canola production in Golestan province of Iran. Nabavi-Pelesaraei et al. [15] analyzed the efficiency of orchardists and identify wasteful uses of energy using DEA for orange production in Guilan province of Iran. Pang el al. [16] studied 87 countries during 2004–2010 in order to examine the effect of clean energy use on total-factor efficiencies under the simultaneous consideration of economic output, energy conservation, and emission reduction. Pardo Martínez and Silveira [17] applied DEA to energy use, energy efficiency, and CO2 emissions in 19 subsectors in the Swedish service sectors during 1993–2008. Ren et al. [18] examined the life cycle energy efficiency of six biofuels in China using DEA. Shi et al. [19] employed DEA with the objective of analyzing the industrial energy efficiency and investigating the maximum energy-saving potential in 28 administrative regions in China. Zha, Zhao and Bian [20] examined with the DEA approach the regional efficiency of energy use and CO2 emissions in China using the 2010 data set. Sun et al. [21] investigated the effects of innovation on the energy efficiency performance for 24 countries for the period 1994–2013. Sun et al. [22] analyzed the environmental performance of 104 countries between 1980 to 2016 using Malmquist-Luenberger productivity index.

In general, it is noticed that sustainable growth models have a robust territorial component. For this reason, when determining territorial units of production, it is needed to maintain efficiency levels above the average. In this way, specific successful practices can be extrapolated to other territories.

However, researches relating the European Funds as instruments to change economic growth models, based on the majority use of petroleum derivatives towards renewable energy sources, are scarce.

Regarding the differences detected in the levels of regional growth in Europe, the contributions of [23,24,25,26,27] are the most relevant. They showed the current economic model trend to increase economic and social inequalities within European regions and stabilize the European Funds’ role. In specific environmental matters, we found the studies of [28,29,30,31,32]. They showed a series of environmental indicators, and they applied their models to analyze specific territories to make a comparison aiming to identify inequalities among regions.

Despite the existing findings in literature, a research linking these two trends has not been found. Specifically, there is no study that links the regional inequalities in environmental matters and European Funds’ strategic use to reduce them. Moreover, even though both the EU 2020 Growth Strategy and the European Funds’ regulations are provided, the competent authorities’ approval of projects that consider the environmental dimension is contemplated.

For the aforementioned reasons, it is necessary to analyze the environmental efficiency in the use of European Funds from a territorial perspective. In this case, the regional level would be the Spanish regions. In addition, the concept of environmental efficiency would be focused on meeting renewable energy production targets.

Identifying the region with the best results in changing the growth model can allow two types of improvements in public programs management. First, introducing changes in the projects’ approval criteria submitted to the open calls to access the Funds as a financing mechanism for the same. Second, creating a database could be considered where the projects have had the best environmental field results.

#### The European Funds as an Instrument of Change towards a Sustainable Growth Model

Among the European Funds that specifically contemplate the environmental dimension in their regulations are the European Regional Development Funds (ERDF), the European Agricultural Funds for Rural Development (EAFRD), the European Social Fund (ESF), and the European Agricultural Development Fund. Rural (EAFRD) [33].

The ERDF program for the 2014–2019 period prioritized, among the projects presented, those that consider an environmental dimension, those aiming to promote both innovation and the development of Small and Medium-Sized Enterprises (SMEs), employment-generating growth, regional mobility, or development. As result, the promoted projects that aim to create SMEs related to ecological innovations, a low-carbon economy, and efficiency in using resources were benefited. This program also supported the creation of companies related to sustainable tourism, culture, and natural heritage. In this way, creating a shared space for innovation, new information and communication technologies, care for the environment, and energy efficiency is guaranteed.

On the other hand, to promote healthy, sustainable and safe transportation mechanisms, these funds can be accessed by sustainable regional or local mobility projects that contemplate actions to reduce air and noise pollutions. Special mention is dedicated to projects related to energy and climate matters. To this end, this program supports investments to promote energy efficiency and supply security in the European Member States. For this reason, it foresees the development of intelligent systems aimed at improving the efficiency of the distribution, storage, and transmission of energy from renewable sources [34].

**Figure 1:**
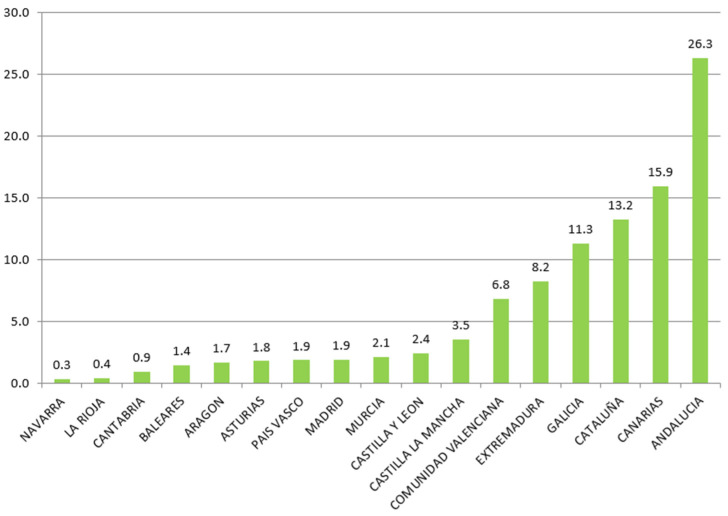
Distribution of ERDF Funds by Region. Period 2014–2019 (%). Source: European Commission and own calculations.

Regarding projects related to urban development, the EU considers it necessary to support initiatives towards the new economic, environmental, climatic, demographic, and social challenges that affect urban areas. Figure 1 shows the distribution of ERDF funds among the Spanish regions for the 2014–2019 period. It shows how the region of Andalusia received 26.3% of the total resources. On the opposite side are La Rioja and Navarra with 0.4% and 0.3%, respectively. 

The ESF sets, among its main objectives, the creation of high levels of quality employment, the improvement in access to the labor market, the promotion of geographical and professional mobility. Lastly, the ESF sets the adaptation of these goals to the industrial changes that occur more and more rapidly in today’s societies. Special reference is made to vulnerable groups. For them, it proposes actions that guarantee a high level of education and training. Likewise, it includes among its objectives, the poverty reduction, social inclusion, gender equality promotion, non-discrimination and equal opportunities.

ESF goals need to consider the support and the creation and modernization of companies. Workers and entrepreneurs must adapt to the new challenges posed by the transition to a knowledge economy, the digital agenda and the shift to a low-carbon and more energy-efficient economy. For this reason, and under the provisions of article 1 of EU Regulation 1303/2013, the training of workers should be given priority to those professional qualifications related to energy efficiency, renewable energies, and sustainable transportation.

**Figure 2:**
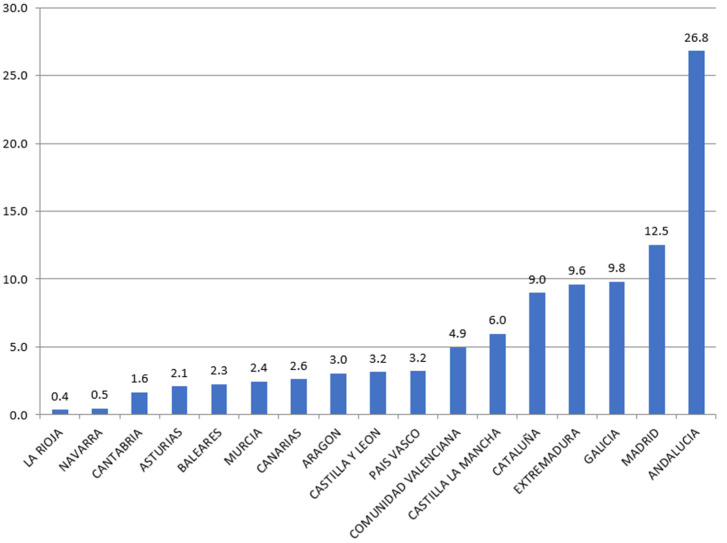
Distribution of ESF Funds by region. Period 2014–2019 (%). Source: European Commission and own calculations.

Figure 2 shows the distribution of the ESF funds among the Spanish regions for the period 2014–2019. It shows how Andalusia (26.8%) and Madrid (12.5%) are the regions that received the most funds during the period. On the contrary, Navarra (0.5%) and La Rioja (0.4%) were the ones that received the least.

The EAFRD contributed to the Europe 2020 Strategy by creating a sustainable rural area throughout the EU. To this end, it financed projects related to the development of the European agricultural sector, linked to creating an increasingly balanced territorial and environmental system. In its implementing regulations, it proposes the achievement, in the long term, of three primary objectives: (a) the promotion of agricultural competitiveness, (b) the sustainable management of natural resources, and (c) action against the climate, and the achievement of balanced territorial development, with the creation and maintenance of jobs.

**Figure 3:**
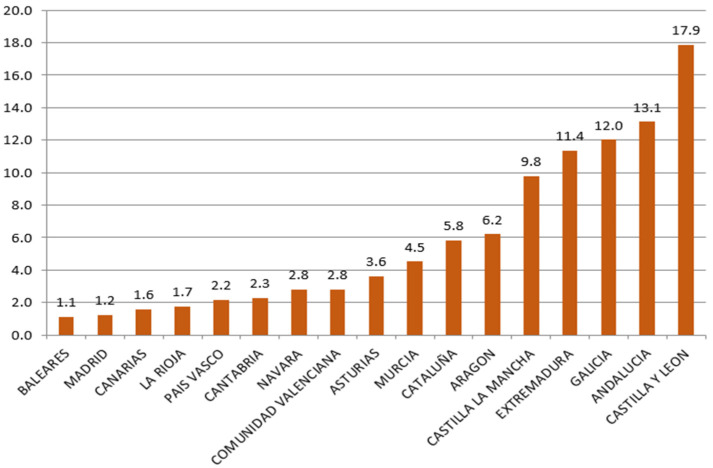
Distribution of EAFRD funds by Region. Period 2014–2019 (%). Source: European Commission and own calculations.

Figure 3 shows the distribution of the EAFRD among the regions for the period 2014–2019. Castilla and Leon (17.9%), Andalusia (13.1%) and Galicia (12%) are the ones that have received the most outstanding amount of resources from the EU. On the contrary, the Region that received the least funds were Madrid (1.2%) and the Balearic Islands (1.1%).

## 3. Methodology

It is necessary to determine a methodology that would allow a comparative efficiency analysis among determined territorial units to fulfill the objective of this research. For this, the bibliographic search was broadened, directing towards parametric or non-parametric models for determining efficiency levels. The results pointed out that an essential part of the published research use the Data Envelopment Analysis (DEA) in determining efficiency levels [12]. Thus, the researches carried out by [12,17,20,35,36,37,38,39,40,41,42,43,44] were considered. Table 1 lists the authors’ primary goals and results obtained with the application of this methodology.

The DEA methodology is based on the model proposed by [35], which allows determining an organization’s relative efficiency concerning others and its distance concerning the efficiency frontier [15]. In this model, an optimal level of efficiency is determined, and it measures the distance between each of the organizations or Decision Making Unit (DMU) concerning it.

In this model, the efficiency of the decision unit (DMU) is obtained as: $Ef=\frac{Y}{X}=\frac{\mathrm{OUTPUT}}{\mathrm{INPUT}}$.

When more inputs are used, the equation would be as follows: $Ef=\frac{a_{i}Y_{i}}{b_{i}X_{i}}$.

The applied model aims to achieve the maximum amount of output given a certain level of inputs, under a restriction of ignorance of the technological level assumed by each DMU. For this reason, the variable-scale returns model (VRS) proposed by Banker, Charles and Cooper is used, oriented towards the output (BBC-output model). Thus, the problem to solve would be the maximization of the following expression: $Max\;y_{j}+\varepsilon \left(\sum_{k=1}^{s}h_{k}^{+}+\sum_{i=1}^{m}h_{i}^{-}\;\right)$.

Subject to: $\sum_{j=1}^{n}\lambda_{j}*x_{ij}=x_{ij}-h_{i}^{-},i=1,\ldots ,m$

(1) $$\sum_{j=1}^{n}\lambda_{j}*y_{kj}=y_{kj}*\gamma_{j}^{}+h_{k}^{+},\;k=1,\ldots ,m$$

(2) $$\sum_{j=1}^{n}\lambda_{j}=1\;\lambda_{j},h_{i}^{-},h_{k}^{+}\geq 0,\forall i,j,k\;\gamma_{j}^{}libre$$

where

$\gamma_{j}^{}$ is the radial enlargement that occurs in all its outputs. It can be identified with the efficiency of j if j is compared with a point belonging to the efficient frontier.

$h_{i}^{-}$ is the rectangular reduction of input i. 

$h_{k}^{+}$ is the rectangular magnification of the output k.

$\lambda_{j}$ represents the coefficients of the linear combination of inputs and outputs to which the DMU projection point is referring on the efficient frontier. It can be interpreted as the proximity of the DMU projection point with respect to the efficient frontier.

In this way, the efficiency frontier would be made up of all those efficient decision units. Once the border has been determined by these entities, it compares each of the entities under study with the border, under the assumption that the detected deviations indicate inefficient behaviors. In this way, the relative efficiency of a set of DMUs that produces a type of output from a common set of inputs can be measured.

In this manuscript, we have used a production function that has an output orientation. Likewise, since there is no certainty about the type of return of the function, a BCC-Output type model has been assumed, which yields a measure of pure technical efficiency. Thus, it was ignored the size of the scale, since it compares only one DMU to a similar scale unit [39].

The defined DMUs are the Spanish regions. From the analysis of the existing literature, the inputs and outputs applied to environmental research that defines a production function, intended to be maximized, are shown in Table 2.

Once the bibliographic analysis has been carried out, the input/output variables used in this research are shown in Table 3.

**Figure 4:**
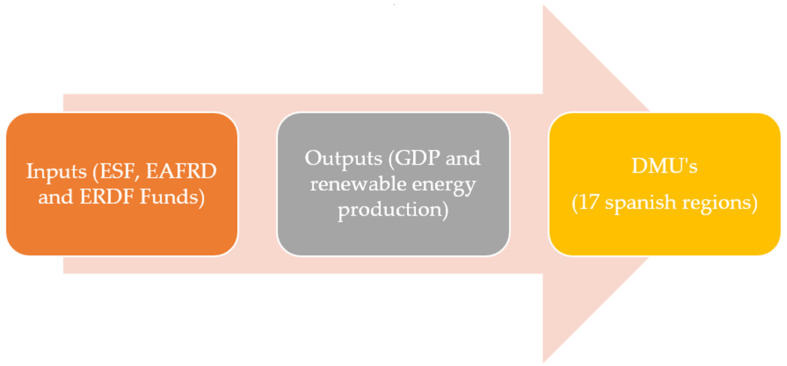
Summary of the applied methodology. Source: Authors’ own elaboration.

Figure 4 shows a summary of the methodology and variables used in this research.

The DMUs used are shown in Table 4.

## 4. Results

### 4.1. DEA Analysis

The analysis of efficiency in using European Funds to improve environmental quality has been carried out through a production function where investments in Funds from the EU would form the inputs variables. The outputs variables would be formed by GDP and energy production through renewable sources. The DMUs used are the Spanish regions. A Data Envelopment Analysis (DEA) has been applied to the function that assumes an orientation towards output (BCC) and variable returns to scale. The results are shown in Table 5. Relative efficiency has been calculated for the years 2014 to 2019 and the mean of the period for each country, the mean of the Spanish regions, and the Spearman correlation coefficient.

Likewise, Table 6 summarizes the average efficiency of the Spanish regions during the period 2014 to 2019. The table shows the number of times the maximum efficiency, the maximum and minimum efficiency found, and the difference between them.

To determine the relations between investments from European funds and efficiency, the total received by each of the Spanish regions and the average level of efficiency for the studied period have been calculated. Subsequently, a simple index was calculated that collects the relations between both variables. 

I_i_ 2014–2019 = Total Funds received by the region i in the 2014–2019 period/Average efficiency obtained by the region i in the 2014–2019 period. 

**Figure 5:**
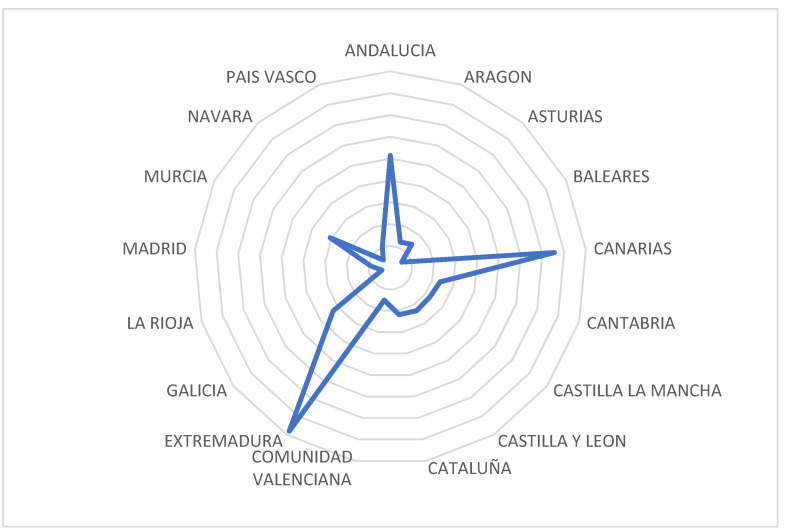
Relations between the studied variables in the Spanish regions 2014–2020 (in euros). Source. Authors’ own elaboration.

The results are shown in Figure 5 as follows.

Figure 5 shows how Extremadura, the Canary Islands, and Andalusia have obtained funds concerning environmental efficiency above the Spanish average.

### 4.2. Results Discussion

The previous literature analysis showed how the current economic model tends to create and amplify regional differences. To reduce these differences, it is necessary to create financial instruments towards the most disadvantaged areas. Specifically, it is needed to highlight the role that the European Funds play in creating a more integrated Europe in the economic, social and environmental fields.

This research shows how this tendency to create inequalities can also be applied to the Spanish territorial energy model. Despite the large amount of economic resources that have come to Spain from Europe, the inequalities were identified. For this reason, it is needed to introduce modifications in the regulation of the Funds. These funds could contribute in a more efficient way to reduce inequalities in the production of renewable energies. Right now, there is an excellent opportunity to put these recommendations into practice by policymakers. Currently, European funds are approved for the period 2021–2027. Five goals have been identified in its distribution: (a) creating an intelligent Europe; (b) more ecological and free of the emission of CO_2_; (c) more connected through the promotion of strategic transportation and the development of digital networks; (d) more social; and (e) closer to the citizens.

The GDP per capita still weighs, obviously, in the distribution of funds. However, new criteria have been included that must be taken into account in the distribution of funds. This distribution would consider youth unemployment rates, the average level of education of the population, the phenomenon of immigration, and the contribution to the fight against climate change. 

For the analyzed period considered in this research, the distribution criterion has been fundamentally the relative regional wealth concerning the EU’s average. Thus, three types of regions have been described. First, the least developed, which is the community of Extremadura. Second, those in the transition phase are Andalusia, Murcia, and Castilla La Mancha. Third, the rest are classified as more developed regions.

Although we highlighted that it is essential to consider each territory’s level of wealth, we believe that meeting specific objectives, among those related to the environment matters, should be given greater weight. Growth is the basis for generating wealth and employment and reducing social differences, but this growth must be subordinated to the respect to the environment.

## 5. Conclusions

This article has analyzed, at regional level, the efficiency in the use of European funds as an instrument to improve the use of renewable sources of energy for the 2014–2020 period. For this, a non-parametric DEA model was used, in which the input variables have been the EAFRD, ERDF, and ESF funds, and the output variables were GDP and the Production of renewable energy.

The results have shown how the level of environmental energy efficiency related to economic growth levels has been very different among the DMUs. These results are in line with those obtained by the researchers in the analyzed bibliography, which show that both pure economic growth and the specific one related to caring for the environment have a very accentuated territorial component. For this reason, it is necessary to analyze the territorial singularities to put in place correction into mechanisms that tend to eliminate the differences. Otherwise, these would increase over time due to economies of scale at the regional level. In this research, the element that can introduce a specific balance dose is the European Funds.

The data from this research place the average Spanish environmental efficiency associated with renewable energy sources at 63.9. Above that average, and in decreasing order, are the following regions: Castilla and Leon, Catalonia, Madrid, Andalusia, Galicia, Aragon, Valencian Community, Castilla La Mancha, and Navarra. Below the average are the Balearic Islands, La Rioja, the Basque Country, Asturias, Extremadura, Murcia, the Canary Islands, and Cantabria. Therefore, these results validate the first working hypothesis that was defined in this research. From the DEA analysis, it can be concluded that there are significant differences in the levels of environmental efficiency related to the use of European Funds.

Subsequently, the Spearman rank correlation coefficient was calculated. This coefficient yields values between −1 and 1. The closer it is to the value 1, it indicates no substantial changes in the regions’ management in terms of efficiency level. It was calculated to compare the relative positions of the periods 2014–2015, 2015–2016, 2016–2017, 2018–2019, and 2019–2020. Except for the period 2016–2017, in all cases, the result was higher than 0.85. In that period, it was somewhat lower (0.68). Therefore, it can be affirmed that the differences that have been revealed in the DEA analysis are maintained throughout the analyzed period.

Finally, an index was calculated to determine the direct relations between the funds received and the efficiency levels. In this case, it was shown that there were no relations between the attributions of European funds and the levels of efficiency achieved by the Spanish regions.

In addition to the recommendation to introduce modifications in the distribution of funds linked to the levels of efficiency achieved by the projects presented to the Spanish regions for approval, it would be recommended that those that have obtained the best results would be published. The publications would create a public system of good practices that can be consulted by those public and/or private entities in charge of presenting and managing projects with an environmental dimension.

## Figures and Tables

**Table 1:** Goals and results of the analyses carried out using the DEA methodology.

Authors	Objective of the Work	Results
Pardo Martínez and Silveira (2012) [17]	To analyze energy use, energy efficiency, and CO_2_ emissions in 19 subsectors in the Swedish service sectors during 1993–2008.	The DEA model results show an increase in technical efficiency and energy efficiency, while there has been a decrease in CO_2_ emissions.
Bian, He, and Xu (2013) [34]	To assess regional energy efficiency in China.	The study results provide some implications for improving energy efficiency and reducing CO_2_ emissions in China.
Ebrahimi and Salehi (2015) [12]	To analyze the pattern of energy use and CO_2_ emission from mushroom production in the Isfahan province of Iran.	They concluded by stating that the optimization of energy use presented an improvement in its efficiency, both in terms of specific energy and net energy.
Lin and Du (2015) [39]	To evaluate China’s regional energy and CO_2_ emissions performance for the period 1997–2009.	Their results were as follows: (1) The Eastern provinces generally performed better than those in the Central and Western areas. (2) Market-oriented reforms, especially factor market promotion, positively affected energy use efficiency and CO_2_ emissions. (3) The share of coal in total energy consumption and the expansion of the industrial sector was negatively correlated with China’s regional performance in energy and CO_2_ emissions.
Song, Hao, and Zhu (2015) [41]	To assess the changes in the transport sector’s environmental efficiency in 30 Chinese provinces between 2003 and 2012.	The authors found that transportation was inefficient in most provinces, and the average environmental efficiency was low (0.45). Overall average efficiency peaked in 2005 and declined continuously to a low in 2009; since then, it has increased. In general, transportation is more efficient in the east than in central or western China.
Suzuki, Nijkamp and Rietveld (2015) [43]	To conduct an efficiency analysis of the energy-environment interface for ten Japanese regions following the Fukushima nuclear power accident.	The results offer a significant contribution to decision making and planning for an efficiency improvement in the energy-environment sector for each region in Japan.
Duan, Guo, and Xie (2016) [37]	To measured the energy and CO_2_ emission performance of thermal power industries in China’s 30 provincial administrative regions during 2005–2012.	They conclude that technological progress is the main driver for improving energy productivity and CO_2_ emissions, working better for the former.
Iftikhar, He, and Wang (2016) [38]	To carry out static and dynamic analysis of energy efficiency and CO_2_ emissions for the leading economies.	The results showed that larger economies with an intensive production strategy, a more extensive secondary industry, and weaker carbon tax laws are more likely to be inefficient.
Suzuki and Nijkamp (2016) [42]	They compared the efficiency of the energy-environmental-economic objectives for the EU, APEC, and ASEAN (A&A) countries, using data sets from 2003 to 2012.	The results showed that the EU countries seem to exhibit generally higher efficiency than the A&A countries.
Tian, Zhao, Mu, Kanianska and Feng (2016) [44]	To analyze the environmental efficiency of China’s open field grape production under the restriction of carbon emissions.	The results indicate that the average environmental efficiency score for grape production in China is at a low level of 0.651. In general, the average environmental efficiencies in the South, Southwest, and Northeast regions are lower than average levels, implying an imbalance in economic production, resource consumption, and environmental efficiency in open field grape cultivation.
Zha, Zhao and Bian (2016) [20]	To evaluated the regional efficiency of energy use and CO_2_ emissions in China using the 2010 data set.	The authors conclude that the uncertainty of CO_2_ emissions has a significant influence on the regional efficiency of energy use and CO_2_ emissions.
Chen and Geng (2017) [36]	To conduct an empirical study of 26 countries of the Organization for Economic Cooperation and Development and Brazil, Russia, India, and China.	Their main conclusion is that there is no significant correlation between the proportion of renewable energy consumption and the performance of saving fossil energy and reducing CO_2_ emissions.
Saglam (2018) [40]	To conduct a Data Envelope Analysis (DEA) to determine the most efficient renewable energy source with predetermined input and output variables, comparing seven primary renewable energy technologies that generate electricity.	The results show that geothermal energy is the most efficient, and solar thermal technologies are the least efficient sources.
Tang, You, Sun and Zhang (2019) [45]	To propose a slack-based parallel measurement model to measure the freight sector’s efficiency in Chinese transport from 2013 to 2017.	The results were the following: (1) There are significant disparities in regional transport efficiency in the cargo sector and its subsectors. (2) The freight sector’s inefficient transport performance is mainly derived from the inland waterways subsector’s poor performance. (3) The volume of cargo and population density positively impacts the rail and road subsectors’ transport efficiency.

**Table 2:** Variables inputs/outputs used by researchers on environmental efficiency analysis.

Author(s)	Input Variables	Output Variables
Pardo Martínez and Silveira (2012) [17]	Capital, Labor, Materials, Energy	Production, the value of service, production in each activity, CO_2_ emissions (undesirable)
Bian, He and Xu (2013) [34]	Labor, Capital, Coal, Oil, Natural gas Non-fossil energy	GDP, CO_2_ emissions (undesirable)
Ebrahimi and Salehi (2015) [12]	Human labor, Diesel fuel, Compost Machinery, Chemicals, Electricity, Water	CO_2_ emission of button mushroom production
Lin and Du (2015) [39]	Capital stock, Labor force, Energy consumption	Gross Domestic Product, CO_2_ emissions (undesirable)
Song, Hao and Zhu (2015) [41]	Labor, Capital, Energy	Added value (desirable), CO_2_ emissions (undesirable)
Suzuki, Nijkamp and Rietveld (2015) [43]	Gross expenditure	Electricity generated, CO_2_ emission
Duan, Guo and Xie (2016) [37]	Electricity generation process	Capital, Labor, Fossil fuel, Auxiliary electricity, Electricity, CO_2_ emissions (undesirable)
Iftikhar, He and Wang (2016) [38]	Labor, Capital, Energy	GDP, CO_2_ emissions (undesirable)
Suzuki and Nijkamp (2016) [42]	Primary energy consumption Population	CO_2_, GDP
Tian, Zhao, Mu, Kanianska and Feng (2016) [45]	Labor, Agricultural film, Diesel Chemical fertilizers, electricity Pesticides, Water, Organic fertilizer	Grapes (desirable), Carbon emission (undesirable)
Zha, Zhao and Bian (2016) [20]	Labor, Capital, Coal, Oil, Natural gas	GDP, CO_2_
Chen and Geng (2017) [36]	Renewable energy, Fossil energy Capital stock, Labor force	Real domestic gross product, CO_2_ emissions
Saglam (2018) [40]	Total system levelized, Cost Land requirement, Water consumption	Plant size, the Capacity factor of each power plant, Employment, Greenhouse gas emissions
Tang, You, Sun and Zhang (2019) [45]	Transportation capacity, Transportation route mileage, Freight turnover volume	Freight turnover volume CO_2_ emissions

Source: Own elaboration.

**Table 3:** The production function of the degree of environmental efficiency.

Type	Variable	Description	Source
Outputs	Oij: GDP at market price	Gross domestic product at the market price of region i in year j	Eurostat
	Oij: Renewable energy production	Electric energy generated using renewable energy from region i in year j. Renewable energy is defined as the contribution of renewable energy to the total primary energy supply (STEP). Renewable energies include the primary energy equivalent of hydroelectric sources (excluding pumped storage), geothermal, solar, wind, tidal, and wave sources. It also includes energy derived from solid biofuels, biogasoline, biodiesel, other liquid biofuels, biogas, and the renewable fraction of municipal waste. This indicator is measured in thousands of toe (tonnes of oil equivalent) and a percentage of the total primary energy supply.	*Red Electrica Española*
Inputs	Iij: European Social Fund	Annual investment in the region I in year j of ESF	European Commission
	Iij: European Agricultural Fund for Rural Development	Annual investment in the region i in year j of EAFRD	
	Iij: European Regional Development Fund	Annual investment in the region i in year j of ERDF	

Source: Own elaboration.

**Table 4:** DMUs used and level of development.

Level of Development	DMUs
Less developed regions	Extremadura
Regions in transition	Castilla La Mancha
	Andalusia
	Region of Murcia
	Canary Islands
More developed regions	Galicia
	Asturias
	Cantabria
	Basque Country
	Navarra
	The Rioja
	Aragon
	Madrid
	Castilla and Leon
	Catalonia
	Valencian Community
	Balearics

Source: European Comission.

**Table 5:** Level of energy efficiency of the regions.

Spanish Regions	2014	2015	2016	2017	2018	2019	Average
ANDALUSIA	91.91	94.35	96.79	100	92.8	100	95.97
ARAGON	41.55	62.83	84.11	94.3	86.67	91.38	76.80
ASTURIAS	19.36	37.71	56.06	38.98	59.98	54.75	44.47
BALEARICS	13.7	56.85	100	60.98	70.16	62.88	60.76
CANARY ISLANDS	20.1	22.17	24.24	19.98	19.9	23.25	21.60
CANTABRIA	6.17	7705	9.24	19.61	27.63	32.64	17.16
CASTILLA LA MANCHA	53.43	72,605	91.78	81.62	67.83	73.45	73.45
CASTILLA AND LEON	100	100	100	100	100	100	100
CATALONIA	100	100	100	100	100	100	100
VALENCIAN COMMUNITY	49.3	74.65	100	100	60.25	58.85	73.84
ESTREMADURA	27.23	26.92	26.61	29.83	26.77	26.16	27.25
GALICIA	82.3	84.02	85.74	70.32	88.15	88.6	83.18
THE RIOJA	6.99	20,975	34.96	100	100	100	60.48
MADRID	99.79	99,895	100	100	100	100	99.94
MURCIA	14.71	19.26	23.81	35.21	27.37	33.56	25.65
NAVARA	18.29	46,325	74.36	100	100	100	73.16
BASQUE COUNTRY	32.08	35,705	39.33	86.91	65.7	61.85	53.59
AVERAGE SPAIN	45.70	56.58	67.47	72.80	70.18	71.02	63.96
SPEARMEN CORRELATION COEFFICIENT BY RANGES		0.90	0.88	0.68	0.82	0.95	

Source: Author’s own elaboration. Note: The number of times of maximum efficiency is the number of times the DMU has been found at the production frontier during the period of time analyzed.

**Table 6:** Basic statistical indicators.

Spanish Regions	Average Period	No. of Times Maximum Efficiency	Efficiency Max	Min Efficiency	Difference
ANDALUSIA	95.98	2.00	100.00	91.91	8.09
ARAGON	76.81	0.00	94.30	41.55	52.75
ASTURIAS	44.47	0.00	59.98	19.36	40.62
BALEARICS	60.76	1.00	100.00	13.70	86.30
CANARY ISLANDS	21.61	0.00	24.24	19.90	4.34
CANTABRIA	17.17	0.00	32.64	6.17	26.47
CASTILLA LA MANCHA	73.45	0.00	91.78	53.43	38.35
CASTILLA AND LEON	100.00	6.00	100.00	100.00	0.00
CATALONIA	100.00	6.00	100.00	100.00	0.00
VALENCIAN COMMUNITY	73.84	2.00	100.00	49.30	50.70
ESTREMADURA	27.25	0.00	29.83	26.16	3.67
GALICIA	83.19	0.00	88.60	70.32	18.28
THE RIOJA	60.49	3.00	100.00	6.99	93.01
MADRID	99.95	4.00	100.00	99.79	0.21
MURCIA	25.65	0.00	35.21	14.71	20.50
NAVARA	73.16	3.00	100.00	18.29	81.71
BASQUE COUNTRY	53.60	0.00	86.91	32.08	54.83
AVERAGE SPAIN	63.96	0.00	72.81	45.70	27.11

Source: Authors’ own elaboration.

## Data Availability

The data presented in this study are available in European Commission.

## References

[1] Wang K., Wei Y.M. (2014). China’s regional industrial energy efficiency and carbon emissions abatement costs. Appl. Energy.

[2] González M.D.C.P., Llorens A.S., Canto M.B. (2015). A regional analysis of active employment policies in the different countries of the Euro Zone. Its evolution in the face of the economic crisis. World Econ. Mag..

[3] Bande R., Fernández M., Montuenga V. (2008). Regional unemployment in Spain: Disparities, business cycle, and wage setting. Labour Econ..

[4] Sánchez-Zamora P., Gallardo-Cobos R. (2019). Diversity, disparity and territorial resilience in the context of the economic crisis: An analysis of rural areas in southern Spain. Sustainability.

[5] Tirado D.A., Díez-Minguela A., Martinez-Galarraga J. (2016). Regional inequality and economic development in Spain, 1860–2010. J. Hist. Geogr..

[6] Yue W., Liu Z., Su M., Gu Z., Xu C. (2021). The impacts of multi-dimension urbanization on energy-environmental efficiency: Empirical evidence from Guangdong Province, China. J. Clean. Prod..

[7] Koçak E., Kınacı H., Shehzad K. (2021). Environmental efficiency of disaggregated energy R&D expenditures in OECD: A bootstrap DEA approach. Environ. Sci. Pollut. Res..

[8] Li X.N., Feng Y., Wu P.Y., Chiu Y.H. (2021). An Analysis of Environmental Efficiency and Environmental Pollution Treatment Efficiency in China’s Industrial Sector. Sustainability.

[9] Ouyang W., Yang J.B. (2020). The network energy and environment efficiency analysis of 27 OECD countries: A multiplicative network DEA model. Energy.

[10] Iram R., Zhang J., Erdogan S., Abbas Q., Mohsin M. (2020). Economics of energy and environmental efficiency: Evidence from OECD countries. Environ. Sci. Pollut. Res..

[11] Banaeian N., Omid M., Ahmadi H. (2012). Greenhouse strawberry production in Iran, efficient or inefficient in energy. Energy Effic..

[12] Ebrahimi R., Salehi M. (2015). Investigation of CO_2_ emission reduction and improving energy use efficiency of button mushroom production using Data Envelopment Analysis. J. Clean. Prod..

[13] Khoshnevisan B., Rafiee S., Omid M., Mousazadeh H. (2013). Reduction of CO_2_ emission by improving energy use efficiency of greenhouse cucumber production using DEA approach. Energy.

[14] Mousavi-Avval S.H., Rafiee S., Jafari A., Mohammadi A. (2011). Improving energy use efficiency of canola production using data envelopment analysis (DEA) approach. Energy.

[15] Nabavi-Pelesaraei A., Abdi R., Rafiee S., Mobtaker H.G. (2014). Optimization of energy required and greenhouse gas emissions analysis for orange producers using data envelopment analysis approach. J. Clean. Prod..

[16] Pang R.Z., Deng Z.Q., Hu J.L. (2015). Clean energy use and total-factor efficiencies: An international comparison. Renew. Sustain. Energy Rev..

[17] Pardo Martínez C.I., Silveira S. (2012). Analysis of energy use and CO_2_ emission in service industries: Evidence from Sweden. Renew. Sustain. Energy Rev..

[18] Ren J., Tan S., Dong L., Mazzi A., Scipioni A., Sovacool B.K. (2014). Determining the life cycle energy efficiency of six biofuel systems in China: A Data Envelopment Analysis. Bioresour. Technol..

[19] Shi G.M., Bi J., Wang J.N. (2010). Chinese regional industrial energy efficiency evaluation based on a DEA model of fixing non-energy inputs. Energy Policy.

[20] Zha Y., Zhao L., Bian Y. (2016). Measuring regional efficiency of energy and carbon dioxide emissions in China: A chance constrained DEA approach. Comput. Oper Res..

[21] Sun H., Edziah B.K., Kporsu A.K., Sarkodie S.A., Taghizadeh-Hesary F. (2021). Energy efficiency: The role of technological innovation and knowledge spillover. Technol. Forecast. Soc. Chang..

[22] Sun H., Kporsu A.K., Taghizadeh-Hesary F., Edziah B.K. (2020). Estimating environmental efficiency and convergence: 1980 to 2016. Energy.

[23] Dall’Erba S., Fang F. (2017). A meta-analysis of the impact of European Union Structural Funds on regional growth. Reg. Stud..

[24] Dall’Erba S., Le Gallo J. (2008). Regional convergence and the impact of European structural funds over 1989–1999: A spatial econometric analysis. Pap. Reg. Sci..

[25] RodrÍguez-Pose A., Fratesi U. (2004). Between development and social policies: The impact of European Structural Funds in Objective 1 regions. Reg Stud..

[26] Armstrong H.W., Giordano B., Kizos T., Macleod C., Olsen L.S., Spilanis I. (2012). The European Regional Development Fund and island regions: An evaluation of the 2000-06 and 2007-13 programs. Isl. Stud. J..

[27] Esposti R., Bussoletti S. (2008). Impact of Objective 1 funds on regional growth convergence in the European Union: A panel-data approach. Reg. Stud..

[28] Agovino M., Casaccia M., Crociata A., Sacco P.L. (2019). European Regional Development Fund and pro-environmental behaviour. The case of Italian separate waste collection. Socio-Econ. Plan. Sci..

[29] Wilkinson D. (1997). Towards sustainability in the European Union? Steps within the European Commission towards integrating the environment into other European Union policy sectors. Environ. Politi..

[30] Trica C.L., Banacu C.S., Busu M. (2019). Environmental factors and sustainability of the circular economy model at the European Union level. Sustainability.

[31] Busu M., Trica C.L. (2019). Sustainability of circular economy indicators and their impact on the economic growth of the European Union. Sustainability.

[32] Promoting Sustainable and Inclusive Growth and Convergence in the European Union (No. 2019/7). https://www.econstor.eu/handle/10419/208040.

[33] Bengochea-Morancho A., Higón-Tamarit F., Martínez-Zarzoso I. (2001). Economic growth and CO2 emissions in the European Union. Environ. Resour. Econ..

[34] Bian Y., He P., Xu H. (2013). Estimation of potential energy-saving and carbon dioxide emission reduction in China based on an extended non-radial DEA approach. Energy Policy.

[35] Cardenete M.A., Delgado M.C. (2015). Análisis del Impacto de los Fondos Europeos 2007-2013 en Andalucía a través de un Modelo de Equilibrio General Aplicado. Investigaciones Regionales. J. Reg. Stud..

[36] Chen W., Geng W. (2017). Fossil energy saving and CO2 emissions reduction performance, and dynamic change in performance considering renewable energy input. Energy.

[37] Duan N., Guo J.P., Xie B.C. (2016). Is there a difference between the energy and CO2 emission performance for China’s thermal power industry? A bootstrapped directional distance function approach. App. Energy.

[38] Iftikhar Y., He W., Wang Z. (2016). Energy and CO_2_ emissions efficiency of major economies: A non-parametric analysis. J. Clean. Prod..

[39] Lin B., Du K. (2015). Energy and CO_2_ emissions performance in China’s regional economies: Do market-oriented reforms matter?. Energy Policy.

[40] Saglam Ü. The Efficiency Assessment of Renewable Energy Sources with Data Envelopment Analysis. Proceedings of the 2018 Annual Meeting of the Decision Sciences Institute Proceedings.

[41] Song X., Hao Y., Zhu X. (2015). Analysis of the environmental efficiency of the Chinese transportation sector using an undesirable output slacks-based measure data envelopment analysis model. Sustainability.

[42] Suzuki S., Nijkamp P. (2016). An evaluation of energy-environment-economic efficiency for EU, APEC, and ASEAN countries: Design of a Target-Oriented DFM model with fixed factors in Data Envelopment Analysis. Energy Policy.

[43] Suzuki S., Nijkamp P., Rietveld P. (2015). A target-oriented data envelopment analysis for energy-environment efficiency improvement in Japan. Energy Effic..

[44] Tian D., Zhao F., Mu W., Kanianska R., Feng J. (2016). Environmental efficiency of Chinese open-field grape production: An evaluation using data envelopment analysis and spatial autocorrelation. Sustainability.

[45] Tang T., You J., Sun H., Zhang H. (2019). Transportation Efficiency Evaluation Considering the Environmental Impact for China’s Freight Sector: A Parallel Data Envelopment Analysis. Sustainability.

